# A Novel Role for the Centrosomal Protein, Pericentrin, in Regulation of Insulin Secretory Vesicle Docking in Mouse Pancreatic β-cells

**DOI:** 10.1371/journal.pone.0011812

**Published:** 2010-07-27

**Authors:** Agata Jurczyk, Steven C. Pino, Bryan O'Sullivan-Murphy, Martha Addorio, Erich A. Lidstone, Philip diIorio, Kathryn L. Lipson, Clive Standley, Kevin Fogarty, Lawrence Lifshitz, Fumihiko Urano, John P. Mordes, Dale L. Greiner, Aldo A. Rossini, Rita Bortell

**Affiliations:** 1 Department of Medicine, University of Massachusetts Medical School, Worcester, Massachusetts, United States of America; 2 Department of Physical and Biological Sciences, Western New England College, Springfield, Massachusetts, United States of America; 3 Department of Physiology, University of Massachusetts Medical School, Worcester, Massachusetts, United States of America; 4 Program in Gene Function and Expression, University of Massachusetts Medical School, Worcester, Massachusetts, United States of America; 5 Program in Molecular Medicine, University of Massachusetts Medical School, Worcester, Massachusetts, United States of America; University of Bremen, Germany

## Abstract

The centrosome is important for microtubule organization and cell cycle progression in animal cells. Recently, mutations in the centrosomal protein, pericentrin, have been linked to human microcephalic osteodysplastic primordial dwarfism (MOPD II), a rare genetic disease characterized by severe growth retardation and early onset of type 2 diabetes among other clinical manifestations. While the link between centrosomal and cell cycle defects may account for growth deficiencies, the mechanism linking pericentrin mutations with dysregulated glucose homeostasis and pre-pubertal onset of diabetes is unknown. In this report we observed abundant expression of pericentrin in quiescent pancreatic β-cells of normal animals which led us to hypothesize that pericentrin may have a critical function in β-cells distinct from its known role in regulating cell cycle progression. In addition to the typical centrosome localization, pericentrin was also enriched with secretory vesicles in the cytoplasm. Pericentrin overexpression in β-cells resulted in aggregation of insulin-containing secretory vesicles with cytoplasmic, but not centrosomal, pericentriolar material and an increase in total levels of intracellular insulin. RNAi- mediated silencing of pericentrin in secretory β-cells caused dysregulated secretory vesicle hypersecretion of insulin into the media. Together, these data suggest that pericentrin may regulate the intracellular distribution and secretion of insulin. Mice transplanted with pericentrin-depleted islets exhibited abnormal fasting hypoglycemia and inability to regulate blood glucose normally during a glucose challenge, which is consistent with our in vitro data. This previously unrecognized function for a centrosomal protein to mediate vesicle docking in secretory endocrine cells emphasizes the adaptability of these scaffolding proteins to regulate diverse cellular processes and identifies a novel target for modulating regulated protein secretion in disorders such as diabetes.

## Introduction

Control of blood glucose levels is largely regulated by the release of insulin from the pancreatic islets of Langerhans. Pancreatic β-cells are professional secretory cells where insulin is packaged, stored in mature secretory vesicles and secreted upon appropriate stimulus. Glucose induced stimulation of insulin secretion is typically divided into two phases. A rapid first phase release of insulin was thought to be mediated by granules docked at the cell surface and primed for release [1]. During the second phase of insulin secretion the reserve pool of granules, further away from the plasma membrane, was mobilized to the membrane for release [2]. More recent reports, however, demonstrate that the early phase of insulin secretion is mediated by both predocked granules and those further away from the plasma membrane [3], [4]. Although insulin granule docking is not essential for secretion [5], it seems to provide a temporal constraint for fusion of secretory granules which are further away from the plasma membrane [3].

As with most types of regulated secretory cells, vesicle trafficking and tethering to the plasma membrane in β-cells are regulated by numerous proteins including those contained in the soluble N-ethylmaleimide-sensitive factor attachment protein receptor (SNARE) and exocyst complexes [6]. SNARE complex assembly is necessary but not sufficient for membrane fusion *in vivo*, and other proteins are required for regulating vesicle exocytosis [7], [8]. Relevant to our work, the centrosomal protein, centriolin, was also shown to ‘scaffold’ vesicle-docking exocyst and vesicle-fusion SNARE complexes during vesicle-mediated cytokinesis [9].

Recently, loss-of-function mutations in another centrosomal gene, human pericentrin, have been causally linked to a severe form of dwarfism and microcephaly [10], [11]. Other suggestive features of MOPD II included diabetes, dyslipidemia and hyperinsulinism [11], [12]. Although the cell cycle defects resulting from pericentrin mutations provide a plausible mechanism for the dwarfism associated with MOPD II, the mechanism linking pericentrin with dysregulated glucose homeostasis and diabetes is unknown.

Pericentrin is a component of pericentriolar material (PCM) that surrounds the two centrioles of a centrosome [13]. The centrosome is an organelle in animal cells that regulates cell cycle progression and also serves as the major microtubule organizing center (MTOC). Pericentrin is expressed as B (360 kDa), A (255 kDa), and S (250 kDa) isoforms [13], [14], [15]. These large molecules interact with numerous proteins and protein complexes, including the γ-tubulin ring complex [16], [17], cytoplasmic dynein [18], protein kinase (PK)A, PKCβII [19], [20], DISC1 [21], and PCM-1 [22]. These interactions provide a molecular ‘scaffold’ for many signaling pathways [23]. The scaffolding PCM proteins are very dynamic in trafficking between the centrosome-bound pool and the cytoplasmic pool [24], yet the functional importance of this cytoplasmic localization is not understood.

Here we identify granular cytoplasmic immunostaining of pericentrin associating with insulin secretory vesicles of mitotically quiescent pancreatic islets. In β-cells, RNAi-mediated depletion of pericentrin caused insulin granule hypersecretion, dysregulation of granule docking at the plasma membrane and abnormalities in glucose tolerance tests *in vivo*. This novel function for centrosomal protein in regulation of insulin secretion illustrates the versatility of these scaffolding proteins to modulate multiple cellular processes and identifies a new level of complexity in the governance of regulated protein secretion.

## Results

### Pericentrin Localizes to the Centrosome and Insulin Cytoplasmic Granules in Pancreatic Islets and Insulinoma Cells

**Figure 1:**
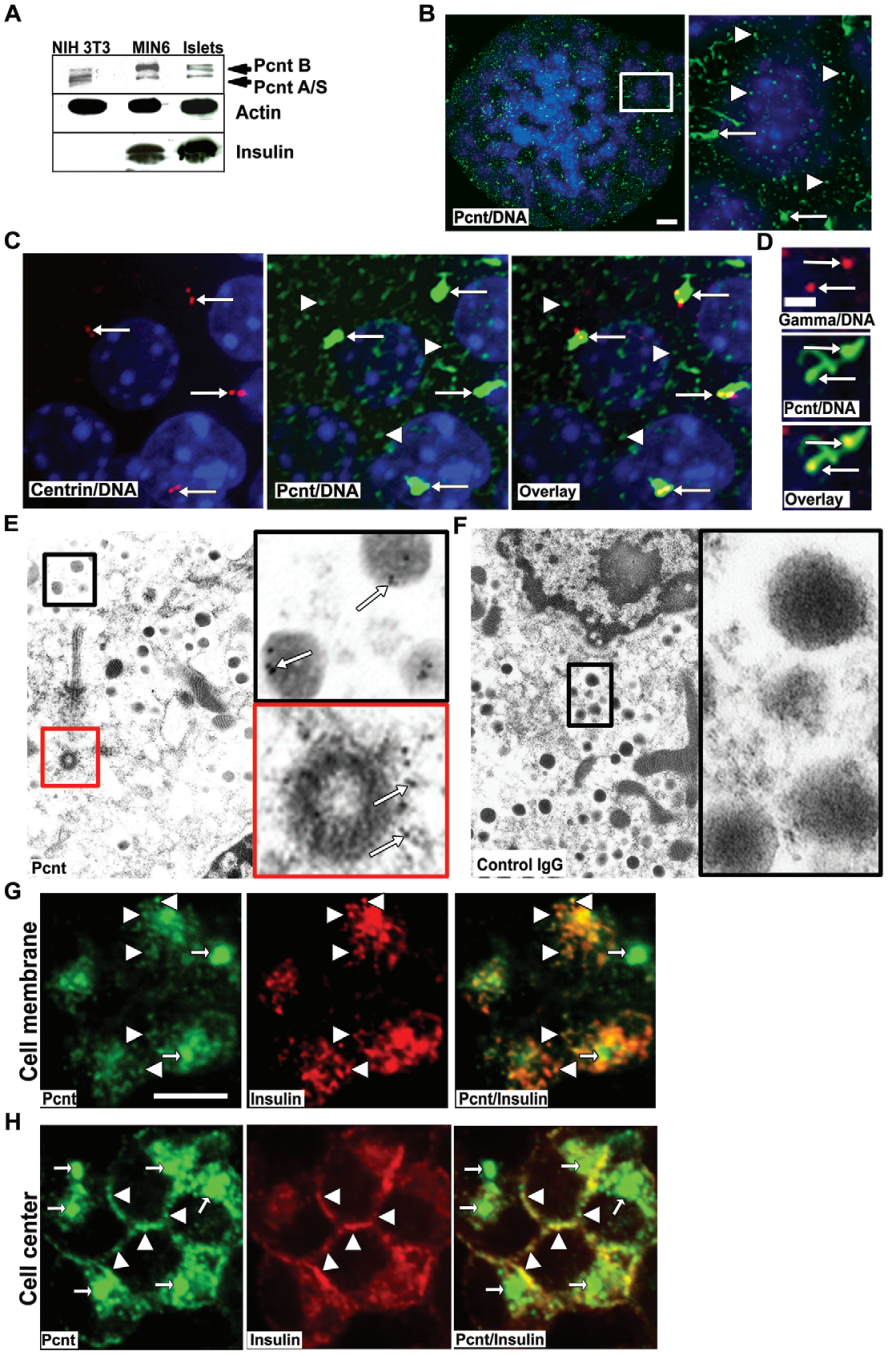
Pericentrin localizes to the centrosome and to secretory vesicles in pancreatic islets. **A.** Western blot of pericentrin (Pcnt) and insulin expression in MIN6, mouse islets and NIH3T3. Actin was used as loading control **B.** Pericentrin (green) localizes to the centrosome (arrows) and cytoplasmic granules (arrowheads) in isolated mouse islets; enlargement of islet cells at right. Cell nuclei are blue (DAPI); scale bar represents 10 µm. **C.** Isolated islets from a GFP-centrin-2 mouse show centrin-2 (red) expression at the centrioles but not visible on cytoplasmic granules. Pericentrin (green) is found on cytoplasmic granules and co-localizes with centrin at the centrosome (overlay in yellow); DNA is blue (DAPI). **D.** Enlargement of the centrosome form mouse islets co-stained with γ tubulin (red) and pericentrin (green) to verify the unusual pericentrin centrosomal staining; scale bar represents 2 µm. **E.** Immuno-EM of isolated mouse pancreatic islets labeled with pericentrin (5 nm gold, arrows). Enlargement shows pericentrin staining on secretory granules (black box) and at the centriole (red box). **F.** Control IgG indicates lack of non-specific staining (enlargement, black box). **G.** Single z-series of pericentrin (green) co-localization with insulin granules (red) at the cell membrane, and **H.** at the cell center.

Although pericentrin is known to be expressed in a wide range of mammalian tissues and transformed cell lines, its expression in the pancreas has not been reported. To gain insight into pericentrin function in differentiated secretory cells, we first analyzed its expression in mouse primary pancreatic islets, as well as transformed mouse insulinoma (MIN6), and NIH 3T3 fibroblast cells. We found all of the pericentrin isoforms (pericentrin B and A/S) to be expressed in fibroblast cells (as previously reported [13]), as well as in professional secretory cells that express high levels of insulin (Figure 1A). Since we did not have specific antibodies to each of the pericentrin isoforms, we could not address their preferential expression. However, based on western analysis (Figure 1A) pericentrin B appears more enriched in the secretory cells than in fibroblasts. Next we examined pericentrin localization within freshly isolated mouse pancreatic islets. We were surprised to find abundant pericentrin staining at both the centrosome and also scattered through the cytoplasm as punctate ‘granules’ (Figure 1B). The filamentous and amorphous appearance of centrosomes was unusual, and prompted us to confirm this morphology using islets isolated from GFP-centrin-2 mice [25]. In these islets we found that GFP-centrin-2 signal localized to typical well-defined centriole doublets, but not to cytoplasmic granules (Figure 1C). In contrast, pericentrin immunostaining of the same islets revealed much larger areas of staining covering both centrioles (consistent with PCM) and prominent granular cytoplasmic staining. We further investigated this atypical centrosome by co-staining with pericentrin and γ tubulin antibodies (Figure 1D); γ tubulin staining was typical of PCM staining, whereas pericentrin staining was more abundant and amorphous. This abundance of pericentrin staining in differentiated islet cells that divide very slowly (∼20 days; [26]) was unexpected given that pericentrin's most recognized function is in regulating cell cycle progression [23]. We further characterized the sites of subcellular localization of pericentrin using immuno-electron microscopy (EM). As expected EM of isolated mouse pancreatic islets showed pericentrin staining at the centrosome (Figure 1E, red box, cross section through a centriole). Suprisingly, cytoplasmic pericentrin was associated with insulin granules, which were identified by their characteristic dense core and surrounding lighter halo (Figure 1E, black box). Control rabbit IgG for the pericentrin affinity-purified antibodies showed no specific staining (Figure 1F).

In mouse insulinoma cells pericentrin co-localized with insulin staining granules along the plasma membrane as shown by single z-sections taken at the ‘top’ (Figure 1G) and through the middle (Figure 1H) of the insulinoma cells. Association of pericentrin with insulin granules from mouse insulinoma cells was also observed by immunofluorescence of purified granules (Figure S1A) and by Western analyses of post-nuclear supernatant fractions (excluding DNA and centrosomes) derived from iodixinol density gradients. Pericentrin co-migrated with proinsulin, insulin, Sec6 (an exocyst component expressed on insulin granules and shown to be important for insulin secretion [27]), SNAP 25 (a SNARE component), and chromogranin B (which is located within secretory granules) (Figure S1B). These results strongly support an association of pericentrin with insulin and other secretory vesicle proteins that constitute the insulin granule.

### Overexpression of Pericentrin Co-Sequesters Insulin Granules with Granular Cytoplasmic Pericentrin

**Figure 2:**
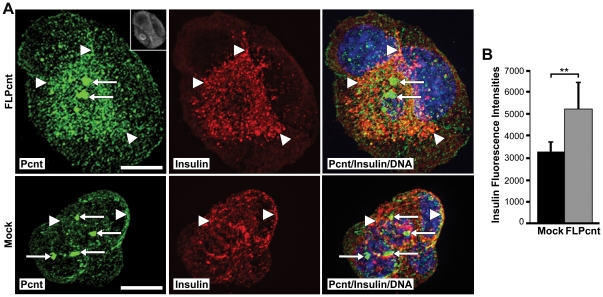
Overexpression of pericentrin in insulinoma cells caused an increase in the granular intracellular insulin staining. **A.** MIN6 cells were transfected with a FLAG-tagged pericentrin expression construct for 48 hr and immunostained with pericentrin (green) and insulin (red) antibodies (FLAG upper right in pericentrin box); arrows indicate the centrosome and arrowheads point to the granular cytoplasmic staining. Yellow shows overlay of pericentrin and insulin immunostaining; nuclei are blue; scale bar represents 10 µm. **B.** Quantitation of insulin fluorescence intensities (** p<0.01). All error bars represent ± SEM. All data shown are representative of multiple independent experiments.

It has been reported previously that pericentrin overexpression in S phase-arrested CHO cells results in formation of a PCM “cloud” consisting of pericentrin and γ tubulin around the multiple centrioles [28]. Our data show that in MIN6 insulinoma cells, overexpression of pericentrin causes an increase in centrosome size (Figure 2A), which could be similar to the PCM “cloud” observed in CHO cells. Increased granular pericentrin staining was also observed in the cytoplasm, and intracellular insulin levels were elevated in pericentrin overexpressing cells as compared to control cells (Figure 2A and 2B). The co-sequestration of pericentrin and insulin with cytoplasmic granules, but not with the centrosomes, further supports close association between insulin granules and cytoplasmic pericentrin staining.

### Pericentrin Depletion Causes a Loss of Intracellular Insulin and Hypersecretion of Mature Insulin without Affecting Proinsulin Biosynthesis

**Figure 3:**
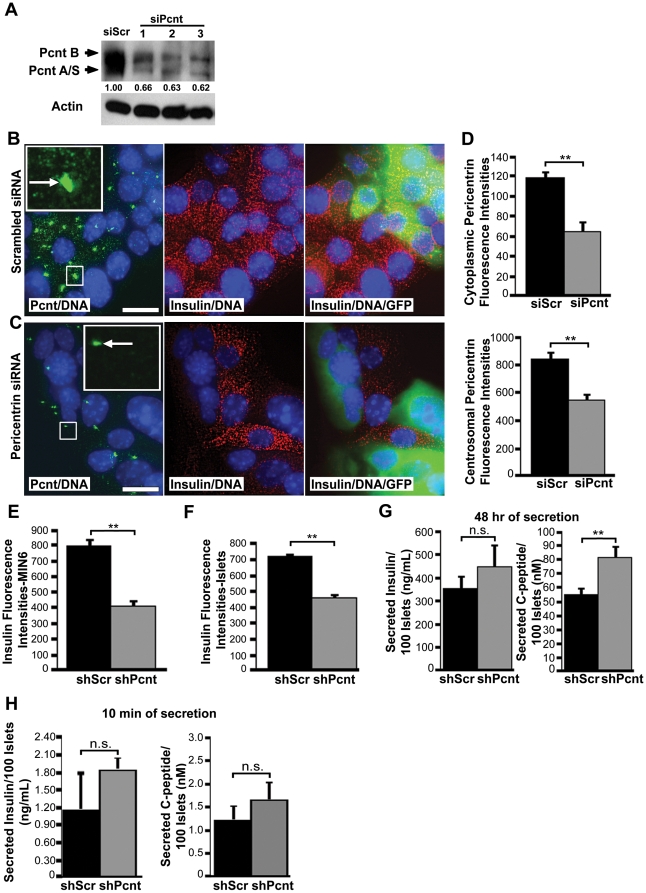
Pericentrin depletion in β-cells causes loss of intracellular insulin and increase in insulin secretion. **A.** Western blot of MIN6 cells treated for 72 h with scrambled (control) and three different pericentrin-specific siRNA. Protein band densities were measured by densitometry and normalized to actin; numbers are relative to siScr control (1.00). **B.** MIN6 cells were treated for 72 h with scrambled siRNA or **C.** pericentin siRNA and immunostained with pericentrin (green) and insulin (red) antibodies as indicated. GFP plasmid was co-transfected for efficiency control. Nuclei are blue (DAPI). Scale bars represent 10 µm. **D.** Quantitation of cytoplasmic and centrosomal pericentrin fluorescence intensities from **B** and **C** (**p<0.01). **E.** Quantitation of cytoplasmic insulin fluorescence intensities from MIN6 cells and **F.** freshly isolated pancreatic islets stably transduced with pericentrin or scrambled shRNA (** p<0.01). **G.** Supernatants from scrambled or pericentrin shRNA-treated islets (48 h of media collection) in culture (n = 3 wells with 100 islets each) or **H.** 10 min glucose stimulated insulin secretion were analysed by RIA for total insulin and C-peptide secretion. (**p<0.01).

To study the function of pericentrin in insulin secreting cells, we depleted pericentrin in pancreatic islets and MIN6 insulinoma cells using RNAi. Western analyses demonstrated pericentrin depletion in insulinoma cells using siRNAs targeting three distinct regions of pericentrin (Figure 3A). Immunofluorescence microscopy revealed centrosomal and granulate staining of pericentrin in scrambled siRNA-transfected cells (Figure 3B). In contrast, pericentrin-specific siRNA-transfected cells showed marked depletion of granular cytoplasmic pericentrin staining (Figure 3C and 3D), and centrosomal pericentrin staining was also diminished as compared to control (siScr) cells (Figure 3C and 3D). Depletion of both cytoplasmic and centrosomal pericentrin staining also confirmed the specificity of the pericentrin antibody. Moreover, transient pericentrin siRNA treatment reduced intracellular insulin content as well (Figure 3B and 3C; a GFP-expressing plasmid was co-transfected as a marker for transfected cells). Stable reduction of pericentrin was achieved using short hairpin RNAs (shRNAs) delivered by pseudotyped lentivirus. This resulted in reduction of intracellular insulin as shown by quantitation of insulin immunofluorescent intensities in insulinoma cells cultured in low (2.5 mM) glucose (Figure 3E).

In order to ensure that the observed reduction of insulin in the insulinoma cells was not due to the known effects of pericentrin depletion on cell cycle progression, we repeated our experiments in quiescent primary islets. We observed similar insulin reductions in freshly isolated mouse islets (Figure 3F and Video S1). The decrease of the intracellular insulin staining correlated with increased insulin secretion into the media from pericentrin-depleted mouse islets cultured at steady state (5 mM glucose) (Figure 3G). Over a 48 h period, pericentrin-depleted islets released more insulin (as measured by C-peptide; Figure 3G). Since C-peptide is a measure of the processed mature insulin, this finding is more important then the non-statistically significant reading of total insulin which consists of both mature/processed and immature/proinsulin. Moreover, 10 min glucose stimulated insulin secretion into the media also seemed to be elevated in pericentrin depleted islets, although this increase was not statistically significant (Figure 3H). This suggests that loss of intracellular insulin seen in pericentrin depleted cells may be the result of hypersecretion.

Having established that increased insulin is secreted into the medium by pericentrin depleted cells, we wanted to address whether loss of intracellular insulin with pericentrin depletion may also be due to effects on insulin synthesis. We performed glucose-stimulated insulin biosynthesis assays with control and pericentrin-depleted cells. Since insulinoma cells are typically cultured in high glucose media (25mM) and have high basal insulin secretion, a series of low non-stimulatory glucose incubations are necessary prior to glucose stimulation. The shRNA-treated insulinoma cells were pre-incubated with 5.5 mM glucose media overnight and then transferred to either low (2.5 mM) or high (25 mM) glucose in the presence of [^35^S]-radiolabeled methionine for a 1 h pulse. We observed the expected increase in [^35^S]-labeled proinsulin with glucose stimulation (Figure S2A). However, there was no difference in proinsulin labeling between control and pericentrin-depleted cells, demonstrating that pericentrin depletion does not appreciably affect insulin biosynthesis. In addition, the amount of insulin in the media in pericentrin depleted cells was increased compared to control cells, again consistent with insulin hypersecretion (Figure S2B). Furthermore, we did not observe any change in cell viability following extended stable depletion of pericentrin up to one month.

### Pericentrin siRNA Depleted Insulin Granules Can Be Restored By Glucose Stimulated Insulin Biosynthesis

**Figure 4:**
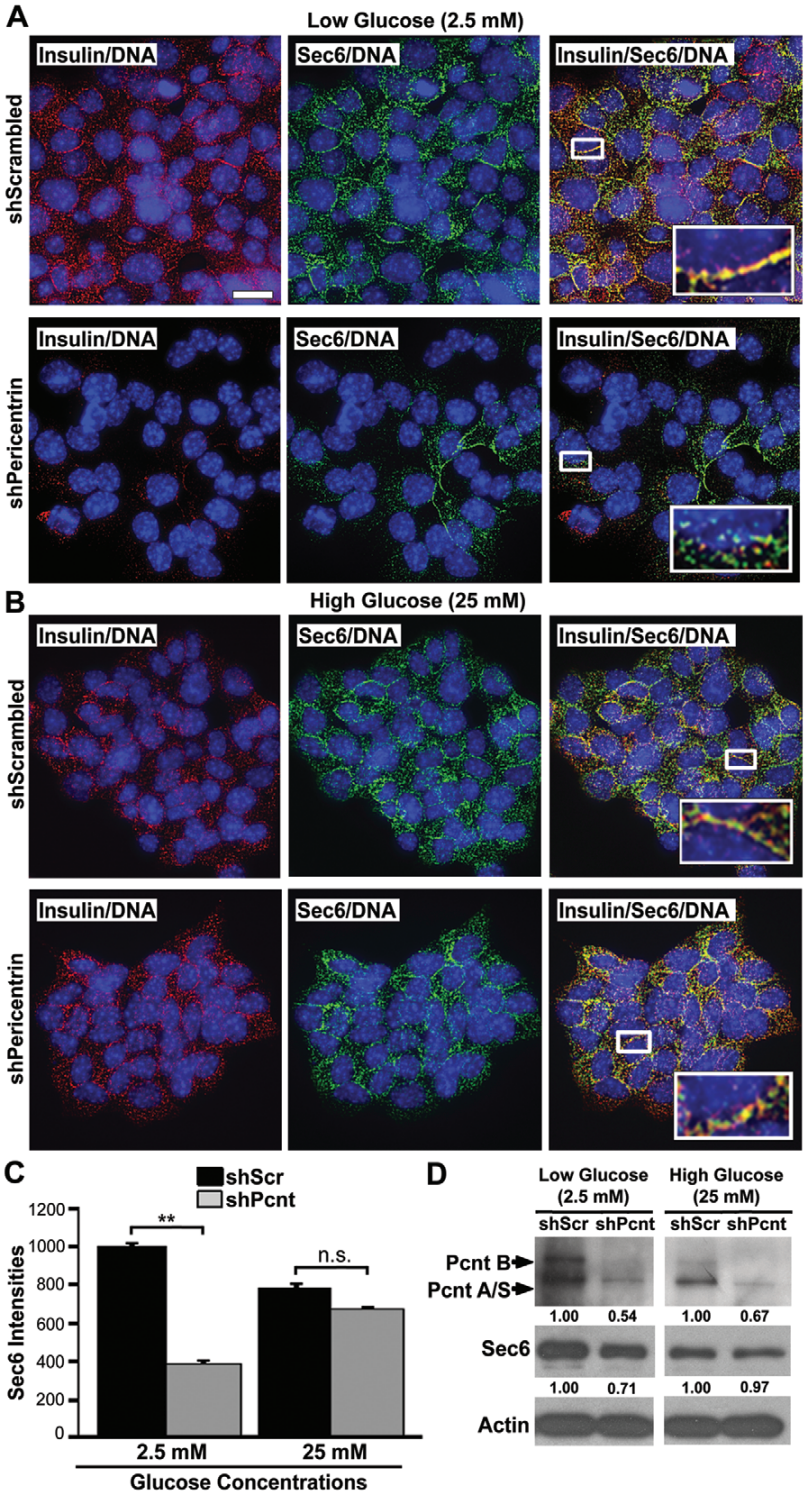
Pericentrin induced loss of insulin granules can be re-established with glucose-stimulated insulin biosynthesis. **A.** Immunofluorescence of shRNA treated stable insulinoma cell lines (both NIT-1 and MIN6 were used with similar results) in low glucose shows co-localization of insulin (red) and Sec6 (green) in scrambled (control) shRNA-treated cells and decreased Sec6 staining and disrupted co-localization with insulin in pericentrin shRNA-treated cells (inset-enlargement). Scale bar represents 10 µm. **B.** Glucose stimulation (25 mM) for 1 hr induced refilling of the insulin granules and return of Sec6 co-localization with insulin in pericentrin-depleted cells (inset-enlargement). **C.** Quantitation of fluorescence intensities for Sec6 from **A** and **B.** **D.** Immunoblot analysis of Sec6 in pericentrin-depleted NIT-1 cells demonstrates reduced Sec6 at low glucose, with a return to normal following high glucose stimulation. Protein band densities were measured by densitometry and normalized to actin; numbers are relative to control (1.00). Data shown are representative of three independent experiments.

In order to determine whether the loss of cytoplasmic insulin in pericentrin depleted cells was due to depletion of insulin from the granules or loss of total secretory granules, we used Sec6 as another marker for insulin granules [27]. Using glucose stimulated insulin secretion assays described above, we showed that insulin levels as well as Sec6 levels were reduced prior to glucose stimulations (Figure 4A and C–D), suggesting that not only insulin, but secretory granules are depleted in pericentrin shRNA treated insulinoma cells. After stimulation with high glucose, however, pericentrin-depleted cells “refilled” their insulin-Sec6 positive granule content to control cell levels (Figure 4B and 4C–D). Similar results were observed with primary mouse islets transduced with pericentrin or scrambled shRNA (Figure S3A–C). Taken together, these data suggest that loss of insulin granules in pericentrin-depleted cells can be reversed by glucose-stimulated insulin biosynthesis. These experiments also indicate that pericentrin-depleted cells are viable since they respond appropriately to glucose stimulation.

### Pericentrin Is Important for Stability of Docked Insulin Granules

**Figure 5:**
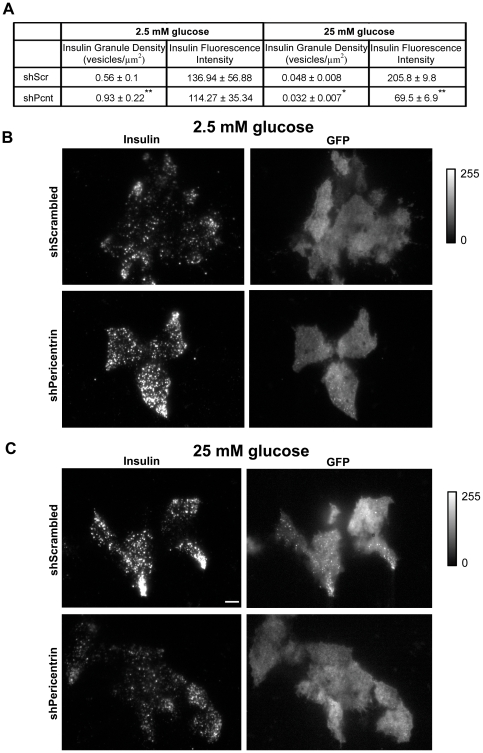
TIRF analyses of pericentrin-depleted MIN6 cells at non-stimulatory glucose concentration (2.5 mM) show increase in docked insulin granules and decrease at stimulatory glucose concentration (25 mM). **A.** Quantitation of insulin granule density and fluorescence intensities before and after glucose stimulation of MIN6 insulinoma cells stably expressing scrambled (control) or pericentrin shRNAs. Values represent means ± s.d; *p<0.05, **p<0.01. All data shown are representative of multiple independent experiments. **B.** TIRF microscopy showing insulin immunofluorescence at the cell membrane (docked granules) before glucose stimulation and **C.** 1 hr after 25 mM glucose stimulation of MIN6 cells stably expressing scrambled or pericentrin shRNAs. GFP reporter indicates shRNA transduced cells. Fluorescence intensity bar to the right, scale bar represents 5 µm.

To continue our investigation whereby pericentrin depletion causes decreased granules and insulin hypersecretion, we next performed total internal reflection fluorescence (TIRF) microscopy. This technology allows visualization of processes within 100 nm of the plasma membrane—and is therefore an effective approach for measuring the pool of ‘morphologically docked’ granules. For these studies we utilized insulinoma cells co-expressing control or pericentrin shRNAs and a transduction marker (GFP). Prior to glucose stimulation (at low glucose), granules should be docked at the membrane as there is no stimulus to release insulin. At low glucose (2.5 mM) concentrations, pericentrin-depleted cells had a statistically higher concentration of docked insulin granules (0.93±0.22) compared to scrambled control (0.56±0.1, p<0.001; Figure 5A and 5B). Following 1 h of glucose stimulation, however, pericentrin depletion resulted in a significant reduction of docked insulin granules (Figure 5A and 5C). In addition to a 1.4 fold decrease in the number of vesicles (shPeri, 0.032±0.007; shScr, 0.048±0.008, p<0.05), there was a large (∼3 fold) decrease in the average insulin intensity of the docked vesicles in the pericentrin-depleted cells (69.5±6.9) compared to scrambled control (205.8±9.8, p<0.001). The fewer remaining vesicles were also dimmer, which indicates that they were either further away from the membrane (defective docking) or had less insulin. However, since pericentrin depletion does not affect on insulin biosynthesis (Figure S2A) we think it is more likely that dimmer vesicles are defectively localized. This increase in membrane-associated vesicles under non-stimulatory conditions followed by fewer residual vesicles after glucose stimulation in pericentrin-depleted cells is consistent with hypersecretion of insulin into the media, as shown in Figure 3G and 3H. Taken together, these data are consistent with a role for pericentrin in regulating the storage and secretion of insulin granules.

### Pericentrin Depletion Does Not Affect Microtubule Nucleation

**Figure 6:**
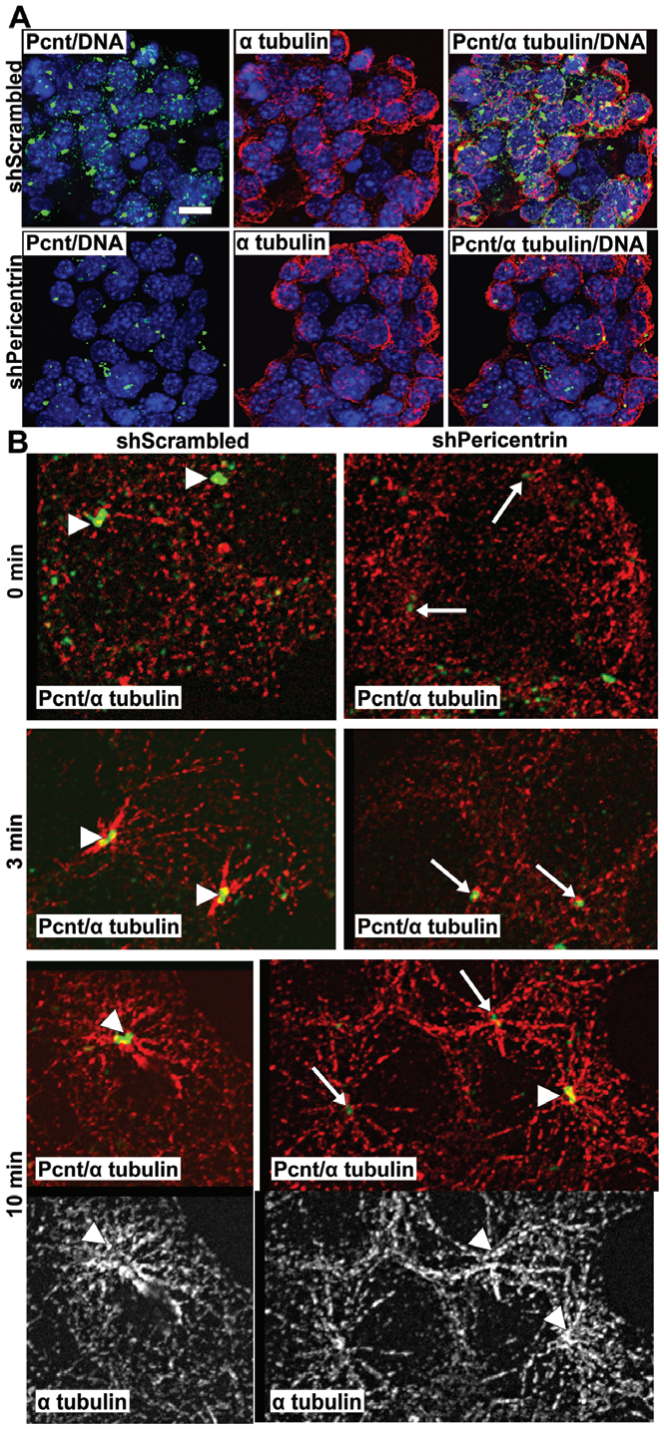
Microtubules are not visibly affected in pericentrin-depleted insulinoma cells. **A.** Immunofluorescence staining for pericentrin (green), α tubulin (red) and DNA (blue) in NIT1 cells stably transduced with scrambled or pericentrin shRNAs; scale bar represents 10 µm. **B.** NIT1 cells were treated with nocodazole (100 µM) for 30 min, and microtubules were allowed to regrow for the indicated times. Arrowheads indicate normal level of pericentrin at the centrosome, arrows indicate depleted pericentrin levels; α tubulin (red or white), pericentrin (green).

Because pericentrin is also a part of the microtubule organizing center (MTOC) and intact microtubules are important for proper insulin secretion [29], we also examined microtubules in shRNA-treated cells. As insulinoma cells do not have a well-defined MTOC, it was difficult to assess microtubule organization (Figure 6A). Therefore, we performed microtubule re-growth assays to determine microtubule nucleation in these cells. Control nocodazole-depleted microtubules were able to re-grow a typical aster by 3 min, whereas pericentrin-depleted cells were delayed slightly (Figure 6B). However, by 10 min the microtubules were fully re-grown in both control and pericentrin-depleted cells. This is in agreement with earlier reports indicating that pericentrin depletion affects mitotic, but not interphase, microtubule organization and nucleation [11], [17]. Further, microtubule-disrupting agents are known to inhibit glucose induced insulin secretion [30], whereas our data demonstrate insulin hypersecretion in pericentrin-depleted cells. Although we cannot rule out small effects of pericentrin depletion on microtubule dynamics, our data argue against a role for pericentrin in microtubule dependent insulin granule trafficking in general.

### Pericentrin Regulates Blood Glucose *In Vivo*

**Figure 7:**
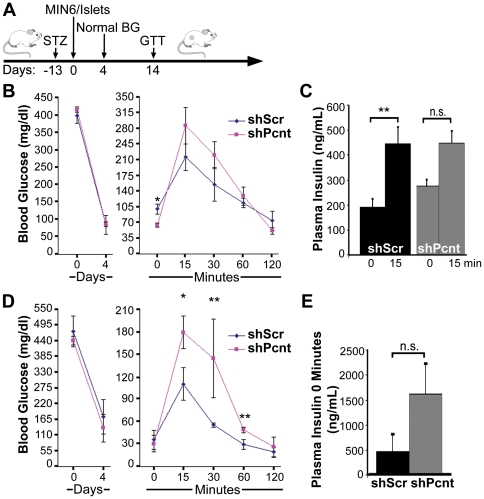
Pericentrin knockdown causes dysregulation of insulin secretion *in vivo*. **A.** Schematic representation of the experimental time course. Streptozotocin (STZ)-induced diabetic mice were transplanted with **B.** syngeneic islets (pericentrin shRNA, n = 3 mice; scrambled shRNA, n = 3 mice) or **D.** MIN6 insulinoma cells (pericentrin shRNA, n = 3; scrambled shRNA, n = 4) which were previously transduced in culture with scrambled or pericentrin shRNAs. After indicated days of transplantation, the mice were fasted and then given a glucose tolerance test (GTT). Blood glucose (BG) was determined at the days and times shown. **C.** Mice transplanted with LV-transduced islets were bled at fasting (time 0) or 15 min following glucose infusion, and plasma insulin levels were determined by RIA. Insulin levels are plotted for one representative experiment (n = 3 per group). For analysis of the change in insulin levels between 0 and 15 min, 2 separate experiments (each with n = 3 per group) were pooled and statistical analysis was based on log transformed data. **E.** Mice transplanted with insulinoma cells were bled at fasting (time 0), and plasma insulin levels were determined by RIA. These graphs show means ± s.e., *P<0.05, **P<0.01.

To examine the significance of pericentrin depletion *in vivo* we transplanted freshly isolated mouse pancreatic islets (or insulinoma cells) transduced with pericentrin or scrambled shRNA into streptozotocin (STZ)-induced diabetic mice (Figure 7A). Islets were transplanted into the renal subcapsular space whereas insulinoma cells were injected subcutaneously. Both scrambled and pericentrin shRNA-transduced pancreatic islets (Figure 7B) or insulinoma cells (Figure 7D) restored normal blood glucose. Two weeks after transplantation, the islet-transplanted mice were fasted prior to administering a glucose tolerance test (GTT). The GTT administered to the mice transplanted with pericentrin-depleted islets showed hypoglycemia after overnight fasting (Figure 7B, time 0). Mice transplanted with the insulinoma cells were only fasted for 5 hrs due to relatively low blood glucose as described previously [31]. The shorter fasting time may explain the failure to observe a difference with pericentrin depletion at time 0 in insulinoma cells, as was observed in the islet transplanted mice fasted overnight. However, similar to the islet transplanted mice (Figure 7C), plasma insulin levels were higher in mice transplanted with pericentrin depleted insulinoma cells compared to control mice (Figure 7E), although the increase was not statistically significant.

After glucose challenge, mice with pericentrin-depleted cells (both islets and insulinoma cells) initially had higher blood glucose levels (Figure 7B and 7D, time 15 min). Plasma insulin levels in mice transplanted with control or pericentrin-depleted islets were both increased at 15 min following glucose injection (Figure 7C). However, because mice with pericentrin-depleted islets had higher fasting insulin levels (Figure 7C, time 0), the change in insulin levels between 0 and 15 min was significantly greater in control than pericentrin-depleted mice. This is consistent with prior depletion of insulin granules, as we observed *in vitro* (Figure 3C). The elevated blood glucose immediately following glucose challenge is also suggestive of inadequate amount of insulin left after insulin hypersecretion in the pericentrin depleted islets or dysfunctional kinetics of insulin granule transit through the cytoplasm. By 120 min, however, blood glucose levels in animals transplanted with pericentrin-depleted islets and insulinoma cells has returned to normal, suggesting that the glucose stimulated insulin biosynthesis and trafficking of granules to the membrane were not inhibited. Intriguingly, the rapid rate of decline (15–120 min) in the blood glucose of pericentrin-depleted mice suggests that glucose-stimulated hypersecretion of insulin may also be occurring *in vivo*, as observed *in vitro* in our glucose stimulated refill of insulin granules experiment (Figure 4).

## Discussion

Mutations in human pericentrin have recently been reported to cause primordial dwarfism and associated clinical disorders including episodes of hyperinsulinemia and early onset of diabetes [11], [12]. To explore a potential role for pericentrin in glucose regulation we investigated its function in insulin-producing pancreatic β-cells. In this study we show that pericentrin is expressed in primary mouse pancreatic islets and insulinoma cells. In addition to its centrosomal localization, we found, unexpectedly, that pericentrin associated with insulin granules. Depletion of pericentrin in β-cells caused a loss of intracellular insulin, decreased the number of Sec6-positive insulin granules docked at the plasma membrane and enhanced insulin secretion. Mice transplanted with pericentrin-depleted islets had abnormal glucose tolerance. These data demonstrate a newly described function for pericentrin to mediate vesicle docking and insulin secretion in β-cells.

To our knowledge, this is the first study to investigate pericentrin expression and localization in pancreatic β-cells, and we demonstrate a novel association of pericentrin with insulin granules in the cytoplasm. Granular cytoplasmic pericentrin localization has been reported previously by Kubo and Tsukita [32] in differentiated, ciliogenic epithelial cells. However, the pericentrin granules in β-cells seem to be distinct from the epithelial cell granules in both size and composition [32]. It is possible that the centrosome-associated pericentrin is also as important for secretion as the cytoplasmic/granule-associated pericentrin since both pools were depleted in our study (Figure 3D).

To investigate the function of pericentrin in β-cells, we performed pericentrin overexpression and RNAi-mediated depletion studies. Consistent with its association with cytoplasmic insulin granules, we found that pericentrin overexpression caused sequestration of intracellular insulin with cytoplasmic, but not centrosomal, pericentrin. Pericentrin depletion on the other hand, resulted in a loss of intracellular insulin and Sec6 expressing granules. Our data further demonstrated that the loss of intracellular insulin in pericentrin-depleted β-cells was not due to inhibition of insulin biosynthesis.

Intact microtubule networks are essential for normal insulin secretion. Given pericentrin's well known roles in microtubule organization we addressed whether intracellular insulin depletion was an indirect effect of pericentrin knockdown using microtubule re-growth assays. We observed no appreciable effect of pericentrin depletion on microtubule nucleation in β-cells, which is not surprising given that other centrosome proteins such as γ-tubulin are still present in pericentrin depleted cells and capable of organizing microtubules networks [33]. However, because microtubule nucleation was somewhat delayed and the intracellular distribution of the insulin granules was transiently affected, we cannot rule out that the positioning and/or transit of insulin granule through the cytoplasm may be altered in pericentrin depleted cells. In addition, we found that both rapidly-dividing insulinoma cells, as well as quiescent primary pancreatic islets, exhibited loss of intracellular insulin with pericentrin depletion, suggesting this phenotype was not secondary to pericentrin-dependent effects on cell cycle progression.

Depletion of granulphilin causes reduced membrane associated vesicles [5], while they are increased in pericentrin-depleted cells. Strikingly, both result in insulin hypersecretion, suggesting that maybe a readiness of these granules for fusion is more important that their actual distribution inside the cell. Pericentrin depletion of β-cells resulted in depletion of intracellular insulin granules following glucose stimulation with corresponding accumulation of insulin in medium. This may occur through inappropriate fusion of the insulin granules with plasma membranes. Since glucose stimulation was able to restore the insulin granules in pericentrin depleted cells we are not anticipating that pericentrin controls vesicle transit to the plasma membrane. The exact mechanism how pericentrin interacts with the vesicle and plasma membrane components to ensure proper insulin secretion is under investigation. However, we speculate that pericentrin may regulate the dynamics of the actin-SNARE interaction which has been reported to be necessary for the final fusion of the vesicle with the plasma membrane, and when disturbed leads to insulin hypersecretion [34]. Similarly, another centrosomal protein, centriolin, has been shown to be important for membrane exocytosis by anchoring exocyst and SNARE complexes during vesicle-mediated cytokinesis [9].

In conclusion, we found that specific RNAi-mediated depletion of pericentrin resulted in consistent phenotypic changes in secretory vesicle docking and dysregulated insulin secretion *in vitro* and *in vivo*. These findings suggest a mechanism by which pericentrin dysfunction could underlie the abnormalities in glucose regulation and early onset of diabetes seen in individuals with MOPD II [11]. We suspect that pericentrin protein scaffolding of secretory granules is a common characteristic of all secretory cells. In support of this, our unpublished observations indicate that pericentrin localizes with secretory vesicles of all the major pancreatic islet cells. Together with the previously published expression of pericentrin in secretory neuronal cells [35], [36], this suggests a functional role for this centrosomal protein in secretion and identifies pericentrin as a novel regulator of exocytosis.

## Materials and Methods

### Ethics Statement

Animals were housed in a viral-antibody-free facility and maintained in accordance with the *Guide for the Care and Use of Laboratory Animals* (Institute of Laboratory Animal Resources, 1996) and guidelines of the University of Massachusetts Institutional Animal Care and Use Committee (IACUC). All research involving animals in these studies was approved by the University of Massachusetts IACUC.

### Antibodies and immunoblotting

Actin, Sec6, chromogranin B, SNAP25 and α tubulin antibodies were from Chemicon International (Temecula, CA); insulin and glucagon were from Dako, Inc. (Carpinteria, CA). Pericentrin mAb was from BD Bioscience and rabbit affinity-purified pericentrin Ab was from S. Doxsey [13]. Horseradish peroxidase (HRP) IgG secondary Abs were from Santa Cruz Biotechnology, and Alexa-Fluor- and Cy5-labeled probes from Molecular Probes and Jackson Immunoresearch, respectively. Actin was used as a loading control for immunoblotting.

### Cell culture and RNAi

Mouse insulinoma (MIN6, NIT1, and TC6) cells were obtained from ATCC (Bethesda, MD). Insulinoma cells were cultured in D-MEM medium (Invitrogen) and isolated islets were cultured on Matrigel-coated plates in CMRL Medium 1066 (Invitrogen). All media were supplemented with 10% FBS, 1 mM sodium pyruvate, 100 U/ml penicillin, and 100 µg/ml streptomycin at 37°C in an atmosphere of 5% CO_2_.

**Transient knockdown with siRNA.** The siRNAs were designed and synthesized at Dharmacon Research (antisense): siPcnt1 AAUCUCUAAAUCUCUCUGCUU, siPcnt2 UUCUCCAUGAUCUCUUUCCUU, siPcnt3 UCUCGCUCCUUCUCUCUCCUU, scrambled (control) 5′ CAGUCGCGUUUGCGACUGG. The Cell Line Nucleofector Kit R and Nucleofector Device (Amaxa Biosystems, Gaithersburg, MD) were used for transfection of cells. Experiments were performed at 72–96 h when knockdown of pericentrin was determined to be maximal.

**Stable knockdown with shRNA.** Stable shRNA constructs were made by insertion of the appropriate sequence into the pLL3.7 lentiviral (LV) vector [37]. Two different shRNAs for pericentrin were used: 1) 5′ GCAGCTGAGCTGAAGGAGA 2) 5′ CGAAGACTTTATCGTAACA. Scrambled shRNA was used as control: 5′ CAGUCGCGUUUGCGACUGG. Transfection of 293FT cells with the LV constructs and packaging plasmids, ΔR8.9 and VSVg, was performed using Lipofectamine 2000 as per manufacturer's instructions (Invitrogen, Carlsbad, CA). LV was harvested at 48 and 72 h post-transfection. The LV supernatant was centrifuged at 120,000× g for 90 min at 4°C, and the concentrated virus was stored at −80°C until use. Concentrated virus was used to infect islets or insulinoma cells. Experiments with primary islets were performed on freshly isolated islets harvested by collagenase digestion as described [38] after 72–96 h LV infection (when pericentrin knockdown was determined to be maximal). Insulinoma cells were FACS-sorted (for the GFP reporter) one week after LV infection, and GFP-positive cells were used for experiments.

### Glucose stimulations

RNAi-treated insulinoma islets or islets were pre-incubated overnight (16–18 h) in 5.5 mM glucose medium and then incubated for 1 h in KRB buffer consisting of 135 mM NaCl, 3.6 mM KCl, 10 mM Hepes [pH 7.4], 5 mM NaHCO_3_, 0.5 mM NaH_3_PO_4_, 0.5 mM MgCl_2_, 1.5 mM CaCl_2_, and 2.5 mM (low) glucose. Stimulation was performed with 25 mM (high) glucose for the indicated periods of time.

**Insulin biosynthesi.** Proinsulin biosynthesis was analyzed by proinsulin immunoprecipitation of [^35^S] methionine-labeled insulinoma lysates as described [39].

**Radioimmunoassay (RIA).** Isolated mouse pancreatic islets were transduced with scrambled or pericentrin shRNAs for 48 h and then transferred to fresh media containing basal glucose (5.5 mM) for an additional 48 h for analysis of insulin secretion. RIA for secreted insulin and C-peptide in the culture supernatants was performed by Linco Diagnostics (St. Charles, MO).

### Mice Pancreatic islet isolation and transplantation

Balb/c mice were obtained from Charles River (Wilmington, MA). Eight- to 15-week-old mice of either sex were used. Mice were rendered hyperglycemic with a single intraperitoneal injection of 170 mg/kg of streptozotocin (Sigma, St. Louis, MO). Diabetes was defined as a plasma glucose concentration >250 mg/dl (Accu-Chek Active meter, Roche Diagnostics, Indianapolis, IN) on two successive days. Mouse pancreatic islets were harvested by collagenase digestion as described [38]. Islets were stably transduced with lentivirus shRNAs and transplanted at a dose of 20/g body weight into the renal sub-capsular space of recipient mice. In some experiments 2×10^6^ MIN6 cells in a volume of 100 µl of Matrigel (BD Pharmingen) solution were transplanted subcutaneously instead of islets.

### Immunofluorescence

Islets were fixed in 3.7% paraformaldahyde for 30 min at room temp. Immunofluorescence was performed as described [9]. Secondary Alexa-Fluor antibodies were used at 1∶1000, Cy-5 at 1∶100, and DAPI at 1∶10,000 (Sigma-Aldrich). Coverslips were mounted with Prolong Antifade medium (Invitrogen). Images were captured using spinning-disk confocal microscopy on a Nikon Eclipse TE2000-E microscope, deconvolved and analyzed using MetaMorph software. Unless otherwise indicated, all immunofluorescence images were two-dimensional projections of three-dimensional reconstructions to ensure that all stained material was visible in two-dimensional images. All cell tracing and recording of average fluorescence intensities were performed on multiple random fields of cells. To determine centrosomal fluorescence intensity, a square was centered on the centrosome and the average intensity per pixel was recorded. For the cytoplasmic pericentrin intensity, the centrosome intensity was subtracted from the total cytoplasmic intensity. For the insulin cytoplasmic intensities, the total cytoplasmic intensity was used (Metamorph software, Molecular Devices, Downington, PA).

### TIRF microscopy

TIRF imaging was performed as described [40]. To observe GFP we used 488 nm laser for excitation and a 510–540 nm filter for emission. Insulin was visualized with a 568 nm laser and 600–640 nm filter for emission. For quantitative analysis, images of insulin immunofluorescence and GFP fluorescence (as proxy for either scrambled or pericentrin shRNA) were analyzed for the number and brightness of insulin granules. Images were first corrected by subtracting the average background fluorescence as determined from a non-cellular region in the images. The GFP image was used to construct a binary-intensity image identifying just the shRNA expressing cells, which was used to mask the insulin image. Individual granules were then identified. The masked insulin image was convolved with a difference-of-Gaussians filter (smaller Gaussian of 0.42 mm diameter at FWHM intensity; larger Gaussian of 0.82 mm diameter at FWHM intensity) designed to enhance spots of fluorescence about the size of insulin granules against their local intensity background. Those pixels that were local 2-dimensional intensity maxima (compared to all 8 neighboring pixels) were identified and their positions and peak intensities (taken from the corresponding position in unfiltered insulin image) were saved. The binary mask from the GFP image was used to estimate the area of the shRNA expressing cells visible in TIRF and their density and the average brightness of the granules was calculated. We observe a little bleed through from the insulin channel into the GFP channel; however, since GFP was only used to identified the transduced cells, this had no effect on our final results.

### Insulin granule isolation

Subcellular fractionation was used to obtain microsomal fraction-containing insulin granules [41]. MIN6 cells were rinsed three times with PBS containing 0.5 µM sodium orthovanadate and scraped into 500 µl of ice-cold hypotonic lysis buffer (10 mM Tris-HCl pH 7.4, 10 mM NaCl, 30 mM MgCl_2_, 50 mM sucrose, 1 mM Na_2_VO_4_, 10 mM Na_4_P_2_O_7_, 10 mM NaF and protease inhibitors) per 10 cm culture plate. After 4–5 min on ice, the sucrose was adjusted to 250 mM and lysates homogenized by passing 10 times through a 25 gauge needle. Centrifugation steps were as follows: 200× g for 10 min, the supernatant was centrifuged 3,000× g for 10 min, the supernatant was centrifuged at 16,000× g for 10 min, finally the supernatant was centrifuged at 100,000× g for 1 h and the pellet containing the microsomal fraction and granules was resuspended in 200 µl of homogenization buffer. The microsomal/granule fraction was spun onto coverslips, fixed in 3.7% paraformaldahyde and immunofluorescence was performed.

### Iodixinol density gradient

TC6 cells were treated for 30 min on ice with nocodazole (5 µg/ml) and cytochalasin B (5 µg/ml) and homogenized (0.25 M sucrose, 20 mM Hepes-KOH, pH 7.2, 90 mM KOAc, 2 mM Mg(OAc)_2_ with protease inhibitors). Homogenization was performed by passing the cell lysate through a 22 gauge needle (6×), 26 gauge needle (6×) and 10× with a Dounce homogenizer. The homogenate was centrifuged at 3,000× g for 10 min and the supernatant was collected (post nuclear supernatant – PNS). The PNS was resuspended in 200 µl of homogenization buffer and layered on top of 21% iodixanol (0.5 ml). A continuous 8–19% iodixinol gradient was layered on top of the PNS. The gradient was spun using a SW41Ti rotor at 160,000× g for 16 h as described in the Opti-Prep Application Sheet and as previously described [42]. The size markers included bovine serum albumin (4.3S) and thyroglobulin (19S).

### EM

For post-embedding immuno-EM, islets were fixed in 4% formaldehyde and 0.05% glutaraldehyde in PBS for 30 min at 4°C and processed by standard methods [43]. Primary antibodies were those used for immunofluorescence. Controls included PBS alone and the appropriate IgG corresponding to the primary antibody used; 5 or 10 nm gold-conjugated secondary antibodies (SPI Supplies, West Chester, PA) were used at a concentration of 1∶50. Sections were examined in a Philips CM10 transmission electron microscope.

### Statistics

Statistical analyses were performed with GraphPad Prism software (Graphpad Software, San Diego, CA). Differences were compared by two-tailed unpaired *t*-tests. Values of *p*<0.05 were considered statistically significant. In one experiment (Figure 7C), a log transformation was used for analysis; however, for the sake of interpretation, the non-log transformed values are presented.

## Supporting Information

Figure S1 Association of pericentrin with insulin granules was observed by immunofluorescence of purified granules and iodixinol density gradients. A. Subcellular fractionation of MIN6 cells. The fraction predicted to contain insulin secretory granules was immunostained for insulin and pericentrin, with overlay in yellow (3rd panel). Enlargement of inset (4th panel) shows the granules are less than 1 µm in size, consistent with the size of insulin granules (∼300–350 nm; [44,45]; scale bar represents 1 µm. B. Iodixinol density gradient of TC6 cells. Aliquots of post-nuclear supernatants (PNS) and gradient fractions were analyzed by immunoblotting with pericentrin (Pcnt), Sec6, SNAP25, chomogranin B, and insulin antibodies recognizing both pro- and mature insulin. Fraction numbers are shown at the top; arrows indicate calculated gradient density in Svedberg units. The experiment was repeated four times with similar results.
(7.37 MB TIF)

Figure S2 Pericentrin depletion caused insulin hypersecretion without affecting glucose-stimulated insulin biosynthesis. A. Blot of insulin immunoprecipitation from insulinoma cells incubated with low (2.5 mM) or high (25 mM) glucose for 1 h in the presence of [S35]-methionine. B. Blot of media from insulinoma cells grown in 25 mM glucose. [S35]-labeled proinsulin was visualized by phosphoimager; pericentrin was visualized by Western blot of cell lysates from parallel experiment.
(2.02 MB TIF)

Figure S3 Glucose stimulation of isolated mouse islets in vitro. A. Islets were stably transduced with pericentrin or control (scrambled) shRNAs. Immunofluorescence staining before glucose stimulations in a low glucose media (2.5 mM) showed the expected depletion of pericentrin (green) and reduction of insulin (red). Scale bar represents 10 µm. B. 1 hr stimulations with high glucose media (25 mM) showed that pericentrin-depleted islets were able to refill their insulin granule content. C. Fluorescence quantitation for intracellular insulin from A and B.
(8.15 MB TIF)

Video S1 Spinning-disk confocal z-series through a mouse islet stably expressing pericentrin shRNA showed GFP expression throughout the mouse islet. The video shows GFP reporter expressed by 54% of the islet cells. DNA (blue) was visualized with DAPI. The z-series show the whole islet at 0.25 µm steps (xy pixels = 0.183 µm).
(1.06 MB MOV)
