# Supplementary figures and images for: A Novel Role for the Centrosomal Protein, Pericentrin, in Regulation of Insulin Secretory Vesicle Docking in Mouse Pancreatic β-cells
Source: PLoS One. 2010 Jul 27;5(7):e11812. doi: 10.1371/journal.pone.0011812 (PMC2910730; doi:10.1371/journal.pone.0011812)

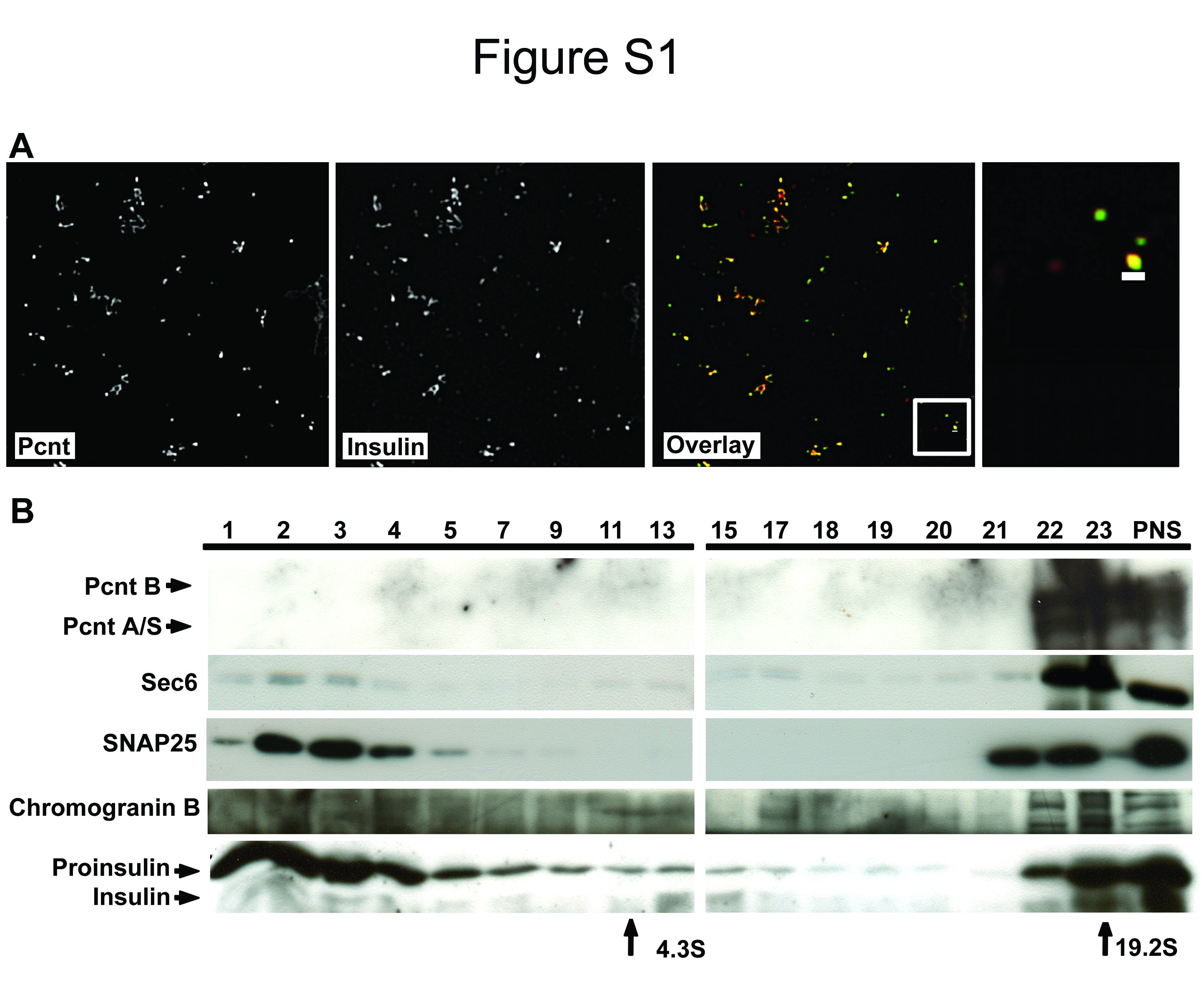

Supplement: Figure S1 — Association of pericentrin with insulin granules was observed by immunofluorescence of purified granules and iodixinol density gradients. A. Subcellular fractionation of MIN6 cells. The fraction predicted to contain insulin secretory granules was immunostained for insulin and pericentrin, with overlay in yellow (3rd panel). Enlargement of inset (4th panel) shows the granules are less than 1 µm in size, consistent with the size of insulin granules (∼300–350 nm; [44,45]; scale bar represents 1 µm. B. Iodixinol density gradient of TC6 cells. Aliquots of post-nuclear supernatants (PNS) and gradient fractions were analyzed by immunoblotting with pericentrin (Pcnt), Sec6, SNAP25, chomogranin B, and insulin antibodies recognizing both pro- and mature insulin. Fraction numbers are shown at the top; arrows indicate calculated gradient density in Svedberg units. The experiment was repeated four times with similar results. (7.37 MB TIF) [file pone.0011812.s001.tif]

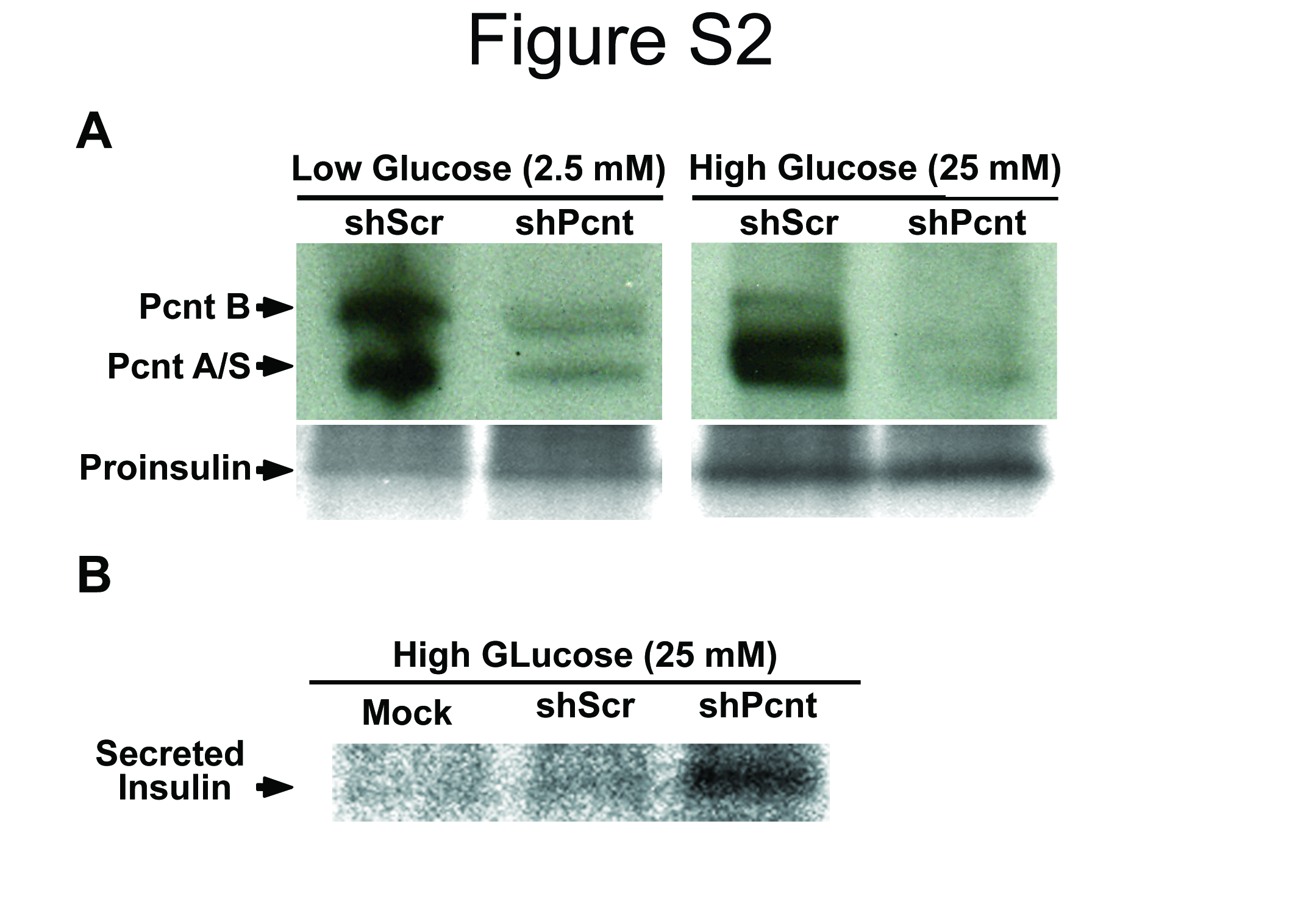

Supplement: Figure S2 — Pericentrin depletion caused insulin hypersecretion without affecting glucose-stimulated insulin biosynthesis. A. Blot of insulin immunoprecipitation from insulinoma cells incubated with low (2.5 mM) or high (25 mM) glucose for 1 h in the presence of [S35]-methionine. B. Blot of media from insulinoma cells grown in 25 mM glucose. [S35]-labeled proinsulin was visualized by phosphoimager; pericentrin was visualized by Western blot of cell lysates from parallel experiment. (2.02 MB TIF) [file pone.0011812.s002.tif]

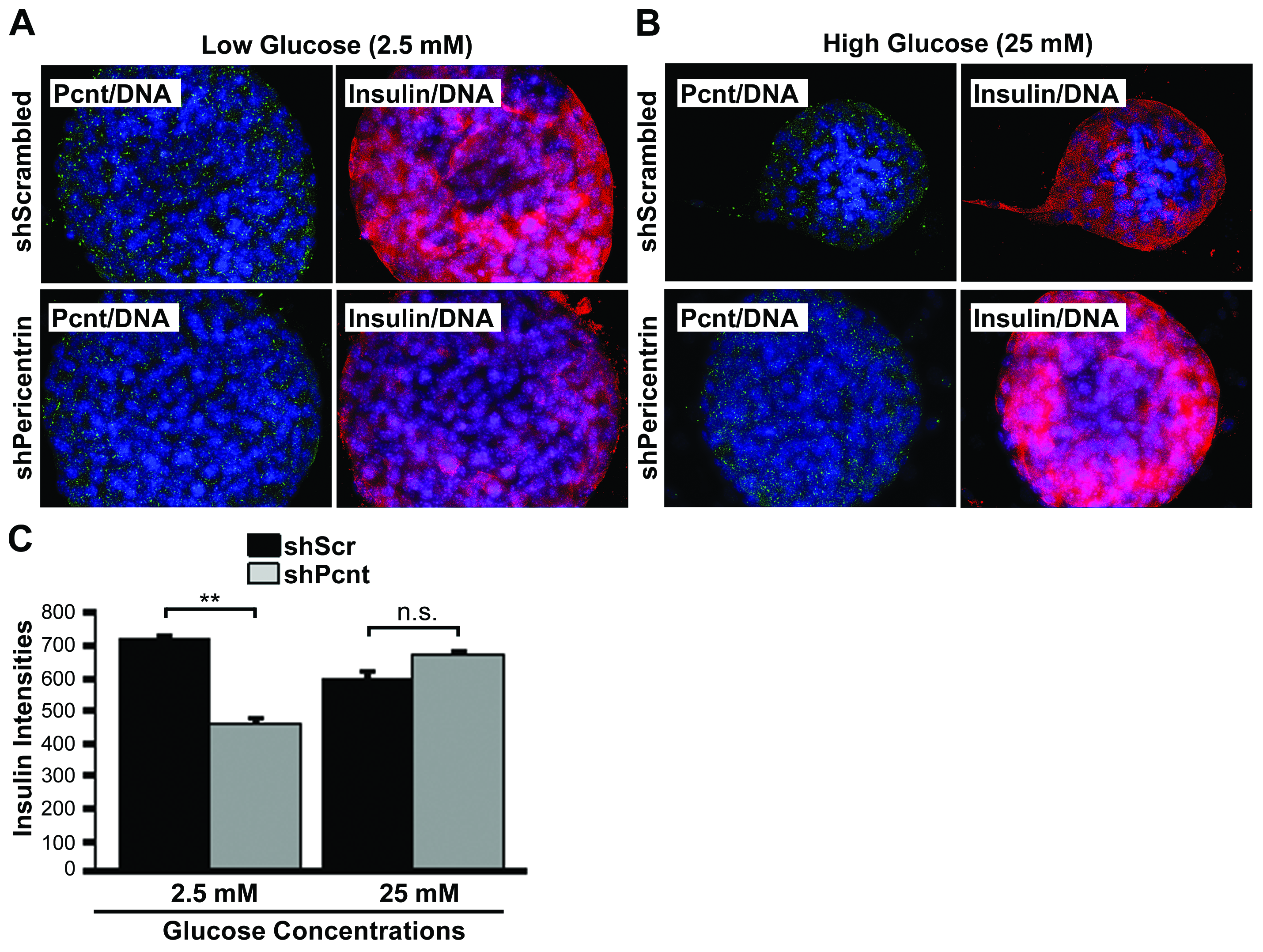

Supplement: Figure S3 — Glucose stimulation of isolated mouse islets in vitro. A. Islets were stably transduced with pericentrin or control (scrambled) shRNAs. Immunofluorescence staining before glucose stimulations in a low glucose media (2.5 mM) showed the expected depletion of pericentrin (green) and reduction of insulin (red). Scale bar represents 10 µm. B. 1 hr stimulations with high glucose media (25 mM) showed that pericentrin-depleted islets were able to refill their insulin granule content. C. Fluorescence quantitation for intracellular insulin from A and B. (8.15 MB TIF) [file pone.0011812.s003.tif]
